# Comparison between two tools assessing the methodological quality of systematic reviews: ReMarQ and AMSTAR 2

**DOI:** 10.1002/gin2.70021

**Published:** 2025-03-29

**Authors:** Manuel Marques‐Cruz, Paula Perestrelo, Alexandro W. L. Chu, Sara Gil‐Mata, Pau Riera‐Serra, Bernardo Sousa‐Pinto

**Affiliations:** ^1^ MEDCIDS‐Department of Community Medicine, Information and Health Decision Sciences; Faculty of Medicine University of Porto Porto Portugal; ^2^ CINTESIS@RISE‐Health Research Network, MEDCIDS, Faculty of Medicine University of Porto Porto Portugal; ^3^ Oncology Department, Local Health Unit of Trás‐os‐Montes e Alto Douro Vila Real Portugal; ^4^ Department of Medicine McMaster University Hamilton Ontario Canada; ^5^ Health Research Institute of the Balearic Islands (IdISBa) Son Espases University Hospital Palma Spain

**Keywords:** AMSTAR, methodological quality, ReMarQ, systematic reviews

## Abstract

Several tools are available for assessing the methodological quality of systematic reviews. The ReMarQ tool – centred on the assessment of the reporting methodological quality of systematic reviews – comprises 26 dichotomous items and does not require clinical or background knowledge of the review topic for its application. In this study, we aimed to compare the results of evaluating the methodological quality of systematic reviews using ReMarQ and A MeaSurement Tool to Assess systematic Reviews (AMSTAR) 2. We assessed a sample of randomly selected systematic reviews published in medical journals using ReMarQ and AMSTAR 2. We calculated the correlation and agreement between the number of fulfilled items in ReMarQ and the number of (i) fulfilled and (ii) fulfilled or partially fulfilled items according to AMSTAR 2. We assessed 51 systematic reviews using both tools. The number of fulfilled items in ReMarQ was strongly correlated with the number of fulfilled items ($r_{s}$ = 0.79; 95%CI = 0.65;0.87) and the number of fulfilled or partially fulfilled items ($r_{s}$ = 0.85; 95%CI = 0.74;0.90) in AMSTAR 2. The percentage of fulfilled ReMarQ items displayed a high agreement with the percentage of fulfilled or partially fulfilled AMSTAR items. In conclusion, the number of fulfilled items in ReMarQ is strongly correlated with that in AMSTAR 2 and there is good agreement between these two tools on the percentage of fulfilled items.

## Key points/Highlights

- The ReMarQ tool assesses the reported methodological quality of systematic review, comprising 26 dichotomous items;
- The number of fulfilled items in ReMarQ strongly correlates with the number of fulfilled items in A MeaSurement Tool to Assess systematic Reviews (AMSTAR) 2;
- ReMarQ and AMSTAR 2 display a strong agreement regarding the percentage of fulfilled items.

Evidence informing guideline recommendations should ideally be based on good quality systematic reviews. Several tools are available for assessing the quality of systematic reviews. The Risk of Bias Assessment Tool for Systematic Reviews (ROBIS) tool is designed to assess the risk of bias in systematic reviews but requires specific clinical or background knowledge of the subject being assessed.^1^ On the other hand, the A MeaSurement Tool to Assess systematic Reviews (AMSTAR) tool is only applicable to systematic reviews of healthcare interventions.^2^ While the original version of AMSTAR was only applicable to systematic reviews of randomised controlled trials, AMSTAR 2 can also be applied to reviews of non‐randomised studies of interventions.^2^, ^3^ However, that still excludes a large number of systematic reviews (e.g., systematic reviews of observational studies quantifying the association between exposures and outcomes or systematic reviews of non‐comparative studies). To overcome these limitations, a new tool – Reporting Methodological Quality (ReMarQ) – has been developed to assess the reporting methodological quality of systematic reviews.^4^ ReMarQ does not require specific clinical or background knowledge of the topic of the review and can be applied to any systematic review. For its development, the authors of ReMarQ consulted tools and guidance documents on methodology (Cochrane Handbook for Systematic Reviews of Interventions^5^), risk of bias (ROBIS^1^) and reporting completeness of systematic reviews (Preferred Reporting Items for Systematic reviews and Meta‐Analysis [PRISMA] statement^6^, ^7^). However, ReMarQ has not been compared to AMSTAR 2 for systematic reviews of intervention studies. Therefore, this study aims to compare the results of assessing the methodological quality of systematic reviews using ReMarQ and AMSTAR 2.

We assessed a random sample of 100 medical systematic reviews using ReMarQ and AMSTAR 2. The eligibility criteria of the systematic reviews and the applied sampling method have been described elsewhere.^4^ In brief, the reviews we assessed represent a random subsample of 400 systematic reviews published between 2010 and 2020 in medical journals indexed in the Journal Citation Reports and were selected using a stratified random sampling approach (Supporting Information: Figure S1). The analysis of a subsample of the 400 systematic reviews was justified on feasibility grounds.

All systematic reviews were assessed using ReMarQ, which evaluates the reported methodological quality of systematic reviews based on 26 dichotomous (“Yes”/“No”) items. Of these, 20 are applicable to all systematic reviews, and six are only applicable to systematic reviews with meta‐analysis (Supporting Information: Table S1). A “Yes” classification indicates that the item was fulfilled (i.e., indicates “good quality on that item”).

Systematic reviews of randomised controlled trials or of non‐randomised studies of interventions were also assessed using AMSTAR 2. AMSTAR 2 includes 16 items, of which 11 are dichotomous (“Yes”/“No”) and 5 can also be answered by “Partial Yes”. We considered a “Yes” classification as indicative that the item was fulfilled (“good quality on that item”) and a “Partial Yes” classification as indicative that the item was partially fulfilled. As with ReMarQ, there are some items in AMSTAR 2 that we only applied to systematic reviews with meta‐analysis (Supporting Information: Table S1). The assessments of systematic reviews using AMSTAR 2 were performed by independent raters who had not evaluated them using ReMarQ and who were blinded to the results of such evaluations.

We calculated the Spearman correlation coefficient ($r_{s}$) between the number of fulfilled items in ReMarQ and the number of (i) fulfilled and (ii) at least partially fulfilled (i.e., fulfilled or partially fulfilled) items according to AMSTAR 2. A sensitivity analysis was performed considering only non‐meta‐analysis‐related questions (i.e., questions that can be applied to all systematic reviews irrespective of whether they have performed meta‐analysis; Supporting Information: Table S1). In addition, to assess the agreement between the percentage of items fulfilled in ReMarQ and AMSTAR 2, we (i) built Bland‐Altman plots, (ii) computed two‐way intraclass correlation coefficients (ICC), and (iii) computed kappa coefficients considering the fulfilment of at least half of the items. We also computed kappa coefficients to assess the agreement of answers to specific individual items that are similar in ReMarQ and AMSTAR 2 (mapping in Supporting Information: Table S1).

In our sample of 100 systematic reviews, we were able to assess only 51 using AMSTAR 2 (Supporting Information: Figure S1). The remaining reviews were excluded because they did not include randomised controlled trials or non‐randomised studies of interventions as their primary studies.

**Figure 1:**
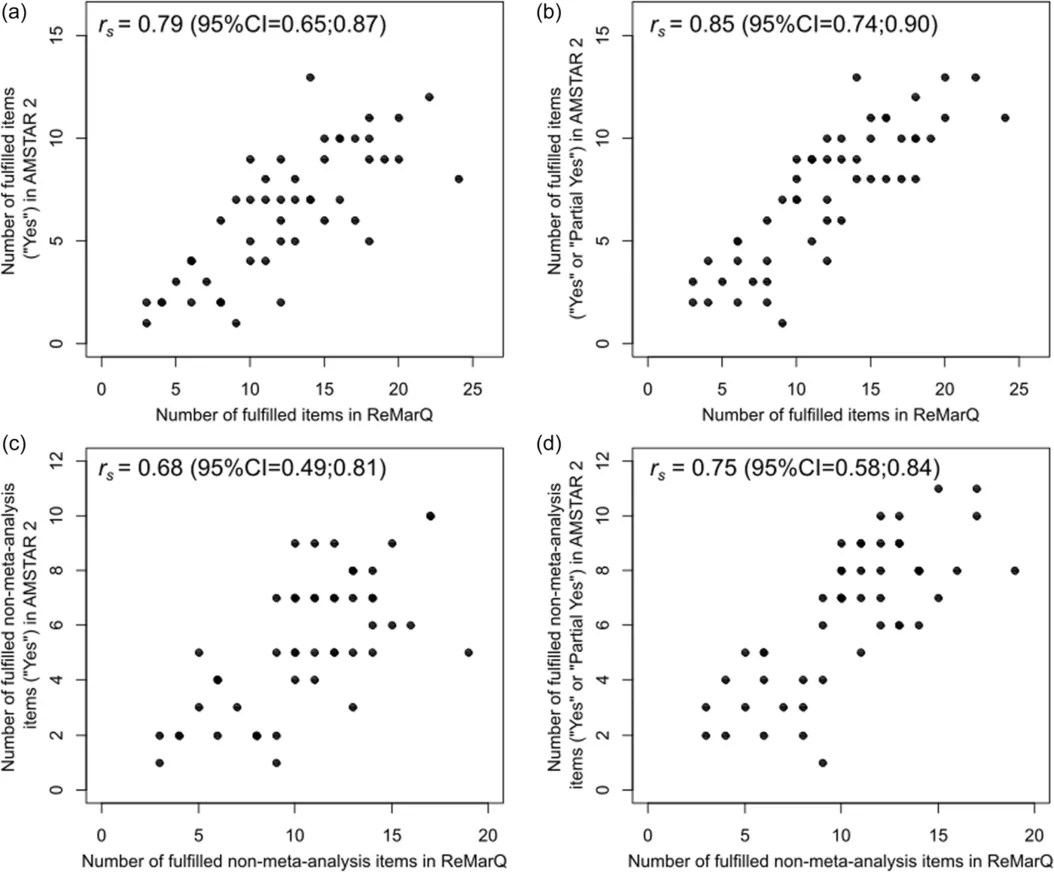
Scatter plots displaying the number of fulfilled items in the reported methodological quality assessment (ReMarQ) tool and the A MeaSurement Tool to Assess systematic Reviews 2 (AMSTAR 2) tool. (a) Scatter plot with the number of fulfilled items in AMSTAR 2 and ReMarQ; (b) Scatter plot with the number of at least partially fulfilled items in AMSTAR 2 and of fulfilled items in ReMarQ; (c) Scatter plot with the number of fulfilled non‐meta‐analysis items in AMSTAR 2 and ReMarQ; (d) Scatter plot with the number of at least partially fulfilled non‐meta‐analytical items in AMSTAR 2 and of fulfilled non‐meta‐analytical items in ReMarQ. CI = Confidence interval; *r*_s_ = Spearman correlation coefficient.

The number of fulfilled items in ReMarQ was strongly correlated with the number of fulfilled items ($r_{s}$ = 0.79; 95%CI = 0.65;0.87) and the number of at least partially fulfilled items ($r_{s}$ = 0.85; 95%CI = 0.74;0.90) in AMSTAR 2 (Figure 1a,b). Strong correlations were also observed when considering only non‐meta‐analysis‐related questions (Figure 1c,d).

**Figure 2:**
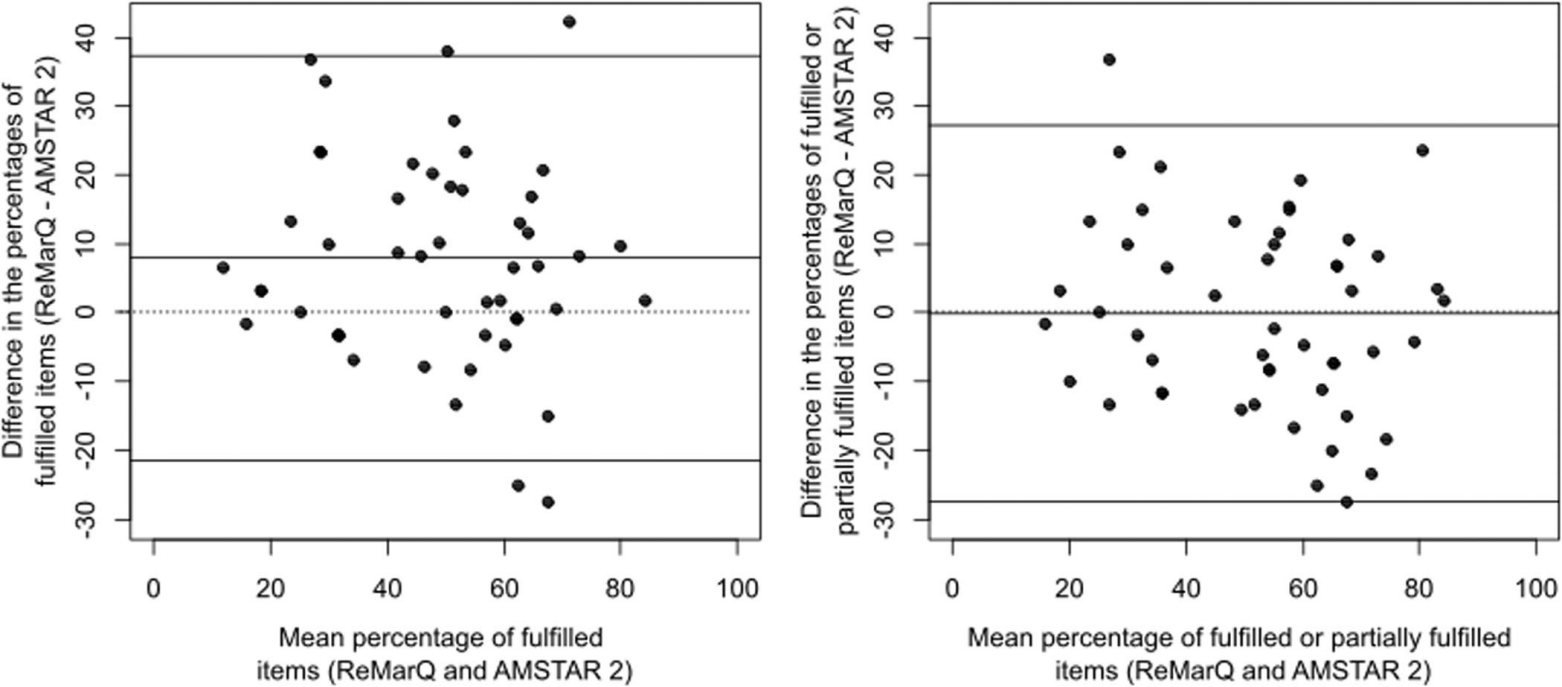
Bland‐Altman plots for the percentage of fulfilled items in the reported methodological quality assessment (ReMarQ) tool and in the A MeaSurement Tool to Assess systematic Reviews 2 (AMSTAR 2) tool.

Regarding the agreement between the percentage of fulfilled items in ReMarQ and the percentage of at least partially fulfilled items in AMSTAR 2, we found a mean difference of −0.2 percent points (pp) (95% limits of agreement = −27.4;27.0 pp) (Figure 2). The ICC was of 0.76 (95%CI = 0.61;0.85). The kappa coefficient for the fulfilment of at least half of the items was of 0.87 (95%CI = 0.73;1.00). Lower agreement was observed with the percentage of fulfilled items in AMSTAR 2 (mean difference of 7.9 pp [95% limits of agreement = −21.3;37.1 pp]; ICC = 0.70 [95%CI = 0.53;0.82]; kappa coefficient for the fulfilment of at least half of the items=0.54 [95%CI = 0.32;0.75]) (Figure 2).

When considering specific individual items that are similar in ReMarQ and AMSTAR 2, the kappa coefficients measuring the agreement of responses ranged to 0.41 (95%CI = 0.09;0.74) to 0.85 (95%CI = 0.69;1.00) (Supporting Information: Table S2).

In this study, we found a strong correlation between the number of fulfilled items according to ReMarQ and the number of at least partially fulfilled items according to AMSTAR 2. Additionally, there was strong agreement between the percentage of ReMarQ fulfilled items and the percentage of AMSTAR 2 at least partially fulfilled items. However, the agreement was not so high for the percentage of AMSTAR 2 (completely) fulfilled items. This discrepancy may be explained by the fact that some questions allowing a “Partial Yes” answer are related to items usually described in the Results section of systematic reviews, whereas ReMarQ is only applicable to the Methods section.

This study has some limitations. Firstly, we were unable to assess half of the systematic reviews in our sample using AMSTAR 2 (due to the designs of the respective primary studies), rendering our estimates less precise. Additionally, assessments were performed by only one reviewer and only once for each review, impairing the evaluation of the intra‐rater and inter‐rater reliability of ReMarQ and AMSTAR 2.

In conclusion, when considering the number of fulfilled items, ReMarQ and AMSTAR 2 display good agreement for systematic reviews of studies of interventions. The dichotomous nature of all its items, and the lack of need for clinical or background knowledge of the topic of the review make the ReMarQ tool a good candidate for large‐scale (or even automated) assessments of the methodological quality of systematic reviews. The results of our study further support such use of ReMarQ.

## AUTHOR CONTRIBUTIONS

**Manuel Marques‐Cruz**: Data curation; formal analysis; methodology; visualization; writing–original draft preparation. **Paula Perestrelo**: Investigation; writing–review and editing. **Alexandro W. L. Chu**: Investigation; writing–review and editing. **Sara Gil‐Mata**: Investigation; writing–review and editing. **Pau Riera‐Serra**: Investigation; writing–review and editing. **Bernardo Sousa‐Pinto**: Conceptualization; investigation; project administration; visualization; writing–original draft preparation.

## CONFLICT OF INTEREST STATEMENT

The authors report no financial conflicts of interest. MMC, PP, SGM and BSP were involved in the development of the ReMarQ tool.

## ETHICS STATEMENT

Not applicable. This study is exempt from ethical committee approval as it consisted in the application of tools to assess the methodological quality of systematic reviews.

## Supporting information

Supporting information.

## Data Availability

The data that support the findings of this study are available from the corresponding author upon reasonable request.
