# Supplementary material for: Comparison between two tools assessing the methodological quality of systematic reviews: ReMarQ and AMSTAR 2
Source: Clin Public Health Guidel. 2025 Mar 29;2(2):e70021. doi: 10.1002/gin2.70021 (PMC13563330; doi:10.1002/gin2.70021)
Supplement: Supplementary file 1 [file GIN2-2-e70021-s001.docx]

**Supplementary Table 1. Mapping of items of ReMarQ and AMSTAR 2.**

| ReMarQ | AMSTAR 2 |
| --- | --- |
| Q1. This systematic review claimed to comply with the PRISMA or other guidelines. |  |
| Q2. A review protocol exists and its registration information was available. | Q2. Did the report of the review contain an explicit statement that the review methods were established prior to the conduct of the review and did the report justify any significant deviations from the protocol? |
| Q3. Inclusion criteria for primary studies were clearly defined. | Q1. Did the research questions and inclusion criteria for the review include the components of PICO? |
|  | Q3. Did the review authors explain their selection of the study designs for inclusion in the review? |
| Q4. No language-based exclusion criteria were defined. | Q4. Did the review authors use a comprehensive literature search strategy? |
| Q5. Information sources (e.g., bibliographic databases) in the search were described. |  |
| Q6. Multiple electronic bibliographic databases were searched. |  |
| Q7. Electronic database searching was complemented with other information sources to identify relevant reports. |  |
| Q8. The identification of unpublished reports was explicitly made possible by the search. |  |
| Q9. The date last searched for information was described. |  |
| Q10. Full electronic search strategy was provided for at least one database. |  |
| Q11. Efforts were made to minimise error in selection of studies, namely by having more than one author independently participated in the study selection process. | Q5. Did the review authors perform study selection in duplicate? |
|  | Q7. Did the review authors provide a list of excluded studies and justify the exclusions? |
|  | Q8. Did the review authors describe the included studies in adequate detail? |
| Q12. Efforts were made to minimise error in data collection by having more than one author independently participating in the data extraction from reports. | Q6. Did the review authors perform data extraction in duplicate? |
|  | Q10. Did the review authors report on the sources of funding for the studies included in the review? |
| Q13. Efforts were made to minimise error in data collection by using a prespecified form for data extraction from reports. |  |
| Q14. Processes for obtaining and confirming data from investigators were described. |  |
| Q15. Efforts to avoid double counting of participants/studies were described. |  |
| Q16. Variables for which data were sought were listed. |  |
| Q17. The risk of bias (or methodological quality) of individual studies was formally assessed using appropriate criteria. | Q9. Did the review authors use a satisfactory technique for assessing the risk of bias (RoB) in individual studies that were included in the review? |
| Q18. Efforts were made to minimise error in risk of bias assessment, namely by having it independently conducted by at least two reviewers. |  |
|  | Q13. Did the review authors account for RoB in individual studies when interpreting/discussing the results of the review? |
| Q19. For procedures involving more than one reviewer, methods for solving disagreements were provided. |  |
| Q20. Methods used to assess certainty (or confidence) in the body of evidence for an outcome (e.g., by application of the GRADE framework) were described. |  |
|  |  |
|  |  |
| A. Meta-analytical summary measures were stated. ^a^ | Q11. If meta-analysis was performed did the review authors use appropriate methods for statistical combination of results? ^a^ |
| B. Meta-analytical models and methods were described. ^a^ |  |
| C. Methods for assessing between-study variation (heterogeneity) and/or inconsistency were described. ^a^ |  |
| D. Assessment of the risk of bias that may affect the cumulative evidence (publication bias or selective reporting) has been performed. ^a^ | Q15. If they performed quantitative synthesis did the review authors carry out an adequate investigation of publication bias (small study bias) and discuss its likely impact on the results of the review? ^a^ |
| E. An intention to perform additional analyses has been reported (e.g., sensitivity or subgroup analyses, meta-regression) even if that not had been done on account of low heterogeneity or insufficient number of primary studies. ^a^ | Q14. Did the review authors provide a satisfactory explanation for, and discussion of, any heterogeneity observed in the results of the review? ^a^ |
| F. Biases in primary studies were addressed in meta-analysis. ^a^ | Q12. If meta-analysis was performed, did the review authors assess the potential impact of RoB in individual studies on the results of the meta-analysis or other evidence synthesis? ^a^ |
|  | Q16. Did the review authors report any potential sources of conflict of interest, including any funding they received for conducting the review? |

AMSTAR: A MeaSurement Tool to Assess systematic Reviews; ReMarQ: Reported Methodological Quality; ^a^ Questions which were only answered if the study performed meta-analysis.

**Supplementary Table 2. Agreement for fulfilment of the most similar items in ReMarQ and AMSTAR 2 ^a^**

| **Topic of the item** | **Item number in ReMarQ** | **Item number in AMSTAR 2** | **Kappa coefficient (95%CI)** |
| --- | --- | --- | --- |
| Protocol availability | 2 | 2 | 0.85 (0.69;1.00) |
| Definition of inclusion criteria | 3 | 1 | 0.41 (0.09;0.74) |
| Study selection performed in duplicate | 11 | 5 | 0.67 (0.47;0.88) |
| Data extraction performed in duplicate | 12 | 6 | 0.69 (0.51;0.88) |
| Risk of bias assessment performed | 17 | 9 | 0.76 (0.57;0.95) |

AMSTAR: A MeaSurement Tool to Assess systematic Reviews; CI: Confidence interval; ReMarQ: Reported Methodological Quality; ^a^ We considered only those questions applicable to all systematic reviews. The kappa coefficient measures the agreement between fulfilment of the items in ReMarQ and at least partial fulfilment of the items in AMSTAR 2.

**Supplementary Figure 1. Flow diagram illustrating the selection of systematic reviews**

AMSTAR: A MeaSurement Tool to Assess systematic Reviews; CI: Confidence interval; NRSI: Non-randomised studies of interventions; RCT: Randomised controlled trials; ReMarQ: Reported Methodological Quality
